# Inter-individual genomic heterogeneity within European population isolates

**DOI:** 10.1371/journal.pone.0214564

**Published:** 2019-10-09

**Authors:** Paolo Anagnostou, Valentina Dominici, Cinzia Battaggia, Alessandro Lisi, Stefania Sarno, Alessio Boattini, Carla Calò, Paolo Francalacci, Giuseppe Vona, Sergio Tofanelli, Miguel G. Vilar, Vincenza Colonna, Luca Pagani, Giovanni Destro Bisol

**Affiliations:** 1 Dipartimento di Biologia Ambientale, Università di Roma “La Sapienza”, Rome, Italy; 2 Istituto Italiano di Antropologia, Rome, Italy; 3 Dipartimento di Scienze Biologiche, Geologiche ed Ambientali, Università di Bologna, Bologna, Italy; 4 Dipartimento di Scienze della Vita e dell’Ambiente, Università di Cagliari, Monserrato, Cagliari, Italy; 5 Dipartimento di Biologia, Università di Pisa, Pisa, Italy; 6 National Geographic Society, Washington DC, United States of America; 7 Institute of Genetics and Biophysics “A. Buzzati-Traverso”, National Research Council (CNR), Naples, Italy; 8 APE Lab, Department of Biology, University of Padova, Padova, Italy; 9 Estonian Biocentre, Institute of Genomics, University of Tartu, Tartu, Estonia; Brigham and Women’s Hospital and Harvard Medical School, UNITED STATES

## Abstract

A number of studies carried out since the early ‘70s has investigated the effects of isolation on genetic variation within and among human populations in diverse geographical contexts. However, no extensive analysis has been carried out on the heterogeneity among genomes within isolated populations. This issue is worth exploring since events of recent admixture and/or subdivision could potentially disrupt the genetic homogeneity which is to be expected when isolation is prolonged and constant over time. Here, we analyze literature data relative to 87,815 autosomal single-nucleotide polymorphisms, which were obtained from a total of 28 European populations. Our results challenge the traditional paradigm of population isolates as structured as genetically (and genomically) uniform entities. In fact, focusing on the distribution of variance of intra-population diversity measures across individuals, we show that the inter-individual heterogeneity of isolated populations is at least comparable to the open ones. More in particular, three small and highly inbred isolates (Sappada, Sauris and Timau in Northeastern Italy) were found to be characterized by levels of inter-individual heterogeneity largely exceeding that of all other populations, possibly due to relatively recent events of genetic introgression. Finally, we propose a way to monitor the effects of inter-individual heterogeneity in disease-gene association studies.

## Introduction

Studying groups subject to barriers to gene flow provides a unique opportunity to understand how inbreeding and drift have shaped the structure of human genetic diversity. A very large number of investigations carried out since early ‘70s has examined the effects of isolation on intra- and inter-population variation in diverse geographical contexts, using genetic polymorphisms varying in mode of inheritance and evolutionary rate [1–5]. Currently, the consequences of isolation may be better studied using genome wide approaches (GWAs), such as those based on single-nucleotide polymorphism (SNP) microarrays, which enable the simultaneous analysis of markers distributed across the human chromosomes. Compared to unilinearly transmitted polymorphisms of mitochondrial DNA and the Y chromosome or to small panels of autosomal loci, GWA approaches make it possible to detect the imprints of isolation left on genomic makeup not only by mutation, but also by recombination [6–14].

In a previous paper, we have compared intra and inter-population measures of genomic variation in a large sampling of European populations in order to understand to what extent the discrete open and isolated dichotomous categories correspond to the way in which their genomic diversity is structured [15]. In this new study, we move our focus to the heterogeneity among genomes within populations. Our results highlight the existence of different and partly unexpected patterns, which shed new light on the genetic structure of population isolates and have implications for disease-gene association studies.

## Materials and methods

### Dataset

Our dataset includes 610 healthy unrelated adult individuals from 28 European populations (Table 1), nine of which with clear signatures of genetic isolation [15–17]. The remaining populations were chosen using the following three criteria: (i) geographic proximity with the isolated populations; (ii) geographic coverage of the European continent; (iii) sample size of at least 10 individuals. Compared to the dataset used by Anagnostou et al. [15], we included five open populations (Belarus, Hungary, Lithuania, Romania and Ukraine) and removed the Cimbrians since it lacked consistent signatures of genetic isolation. Despite its limits [15], we maintain here the dichotomy between open and isolated population for practical reasons (see also the Discussion section).

**Table 1:** Demographic information about the populations under study.

POPULATION	LABEL	N	CURRENT CENSUS	TIME SINCE ISOLATION (years before present)	ISOLATION FACTOR	REFERENCE
**North Eastern Italian isolates**						
Sappada	SAP	24	1,307*	~1000	G/L	[15]
Sauris	SAU	10	429*	~800	G/L	[15]
Timau	TIM	24	500*	800–1000	G/L	[15]
**Sardinians isolates**						
Benetutti	BEN	25	1,971*	~5000	G/L	[15]
Carloforte	CFT	25	6,301*	268	G/L	[15]
North Sardinia	NSA	25	96,448*	3900–2900	G/L	[15]
Sulcis Iglesiente	SGL	23	128,540*	2800	G/L	[[15]
**European isolates**						
Orkney	ORK	15	21,349*	~1300	G	[18]
French Basques	BAS	24	~650,000**	5500–3500	G/L	[18]
**South Europe**						
Albania (Gheg)	ALB	24	2,831,741*	-	-	[19]
Croatia	CRO	20	4,284,889*	-	-	[20]
Greece	GRE	20	10,815,197*	-	-	[21]
Spain	SPA	34	46,815,916*	-	-	[21]
**East Europe**						
Belorussia	BEL	17	9,498,700*	-	-	[20]
Bulgaria	BUL	31	7,202,198*	-	-	[21]
Hungary	HUN	19	9,830,485*			[20]
Lithuania	LIT	10	2,842,412*	-	-	[20]
Poland	POL	32	38,511,824*	-	-	[21]
Romania	ROM	16	19,511,000*	-	-	[20]
Russia	RUS	25	144,192,450*	-	-	[18]
Ukraine	UKR	20	42,539,010*	-	-	[22]
**North Europe**						
Norway	NOR	18	5,214,890*	-	-	[21]
British Isles	GBR	16	63,181,775*	-	-	[21]
**West Europe**						
France	FRA	28	67,264,000*	-	-	[18]
**Italy**						
North Italy (Aosta)	NIT	22	34,619*	-	-	[15]
Central Italy (Piana di Lucca)	CIT	25	394,318*	-	-	Tofanelli S., personal communication
South Italy	SIT	18	14,184,916*	-	-	[21]
Sicily	SIC	20	5,077,487*	-	-	[21]

* National population and housing census—2011 (ALB, BEN, CIT, CFT, CRO, CVV, GBR, GRE, NIT, NSA, ORK, POL, SAP, SAU, SGL, SIC, SIT, SPA, TIM)—2014 (BUL)– 2015 (ROM, RUS, NOR)—2016 (BEL, FRA, HUN, UKR)—2017 (LIT)

** EuskoJaurlaritza 2008

### Data analyses

The samples genotyped with the GenoChip 2.0 array were merged with literature data and then filtered according to the standard genotype quality control metrics using PLINK [23]: (i) SNP genotyping success rate > 90%; (ii) individuals with a genotyping success rate > 92%; (iii) absence of relatedness to the 3rd generation (Identity by Descent, IBD > 0.185). Concerning the latter analysis, when a related pair of individuals was detected, only one sample was randomly chosen and used for the subsequent analysis. We also excluded three SNPs which showed a statistically significant departure from Hardy-Weinberg equilibrium (p-value threshold of 1x10^-6^).

Principal components Analysis was performed using PLINK package (v. 1.9). The position of the centroid for each population was identified by averaging the values for the two axes, while the distance of each point from the centroid was calculated using the formula $\sqrt{{\left(\underline{x}-x\right)}^{2}}+\sqrt{{\left(\underline{y}-y\right)}^{2}}$.

The following statistics were also calculated using PLINK package (version 1.9): (i) the proportion of homozygous loci (HOM) for each individual; (ii) the proportion of identical genotypes between pairs of individuals within each population (IBS); (iii) the number and total length of stretches of contiguous homozygous genotypes, RoH-KB and RoH-NSEG, respectively. For the HOM, RoH-KB and RoH-NSEG statistics, the median was taken as a population value, while individual IBS values were then calculated as the mean of each distribution. The RoHs were identified using default settings (sliding window of 5 Mb, minimum of 50 SNPs, one heterozygous genotype and five missing calls allowed), with a minimum-length cut-off of 500 kb and 14 homozygous SNPs [11]. To measure the spread between individual values within each population, the standard sample variance formula was used for all the above parameters.

We used SHAPEIT v2.r790 [24] to phase the data, using the 1000 Genomes dataset as a reference panel. We split our dataset by chromosome and phased all individuals simultaneously and used the most likely pairs of haplotypes (using the—output-max option) for each individual for downstream applications. For the phasing and conversion, we used genetic map build 37 downloaded with SHAPEIT. We painted each individual using every other individuals of the same population as a donor [25]. We first inferred the global mutation probability and the switch rate for chromosomes 1, 5, 8, 12, 17 and 22 in 10 iterations of the EM (expectation maximization) algorithm. We fixed the parameters estimated from this analysis (Ne, -n flag, and θ, -M flag) to infer the ChromoPainter coancestry matrix for each chromosome. Using ChromoCombine, we combined the data into a single final coancestry matrix. The haplotype chunks and their total length were estimated using as recipients and donors the individuals of the same population (CHR_P).

The comparison of inter-individual heterogeneity for measures of intra-population variation as well as CHR_P was estimated through the equality of variances (Brown-Forsythe Levene type procedure), after the application of Bonferroni correction (R package lawstat).

Maximum likelihood estimates of individual ancestries were obtained using ADMIXTURE v1.23 under default values. Its algorithm is relatively robust to SNP ascertainment bias [26] since it assigns individual ancestry to a finite number of population clusters, and uses a large multilocus dataset, while the most informative SNPs for ancestry inference are variants with large frequency differences across populations [27]. We applied unsupervised clustering analysis to the whole sample set, exploring the hypothesis of K = 2 to 15 clusters. Five independent replicates were run and aligned with CLUMPP. Best K was estimated by the cross-error estimation implemented in ADMIXTURE. We calculated individual heterogeneity (ADX_HET) as the squared difference between each ancestry proportion and its population mean, averaged over all possible ancestries. Population heterogeneity was obtained as the median of individual values.

Admixture dates were inferred using the number of ancestry switches and ancestry proportions following Johnson et al [28]. Phased chromosomes were used to run the RFMix algorithm [29] with the PopPhased option and default parameters. This modelling approach identifies the ancestry of discrete genomic segments of arbitrary size using a conditional random field parameterized by random forests trained on a reference population panel. Finally, the output of RFmix was employed to calculate both the number of ancestry switches and ancestry proportions for each target individual.

## Results

**Fig 1:**
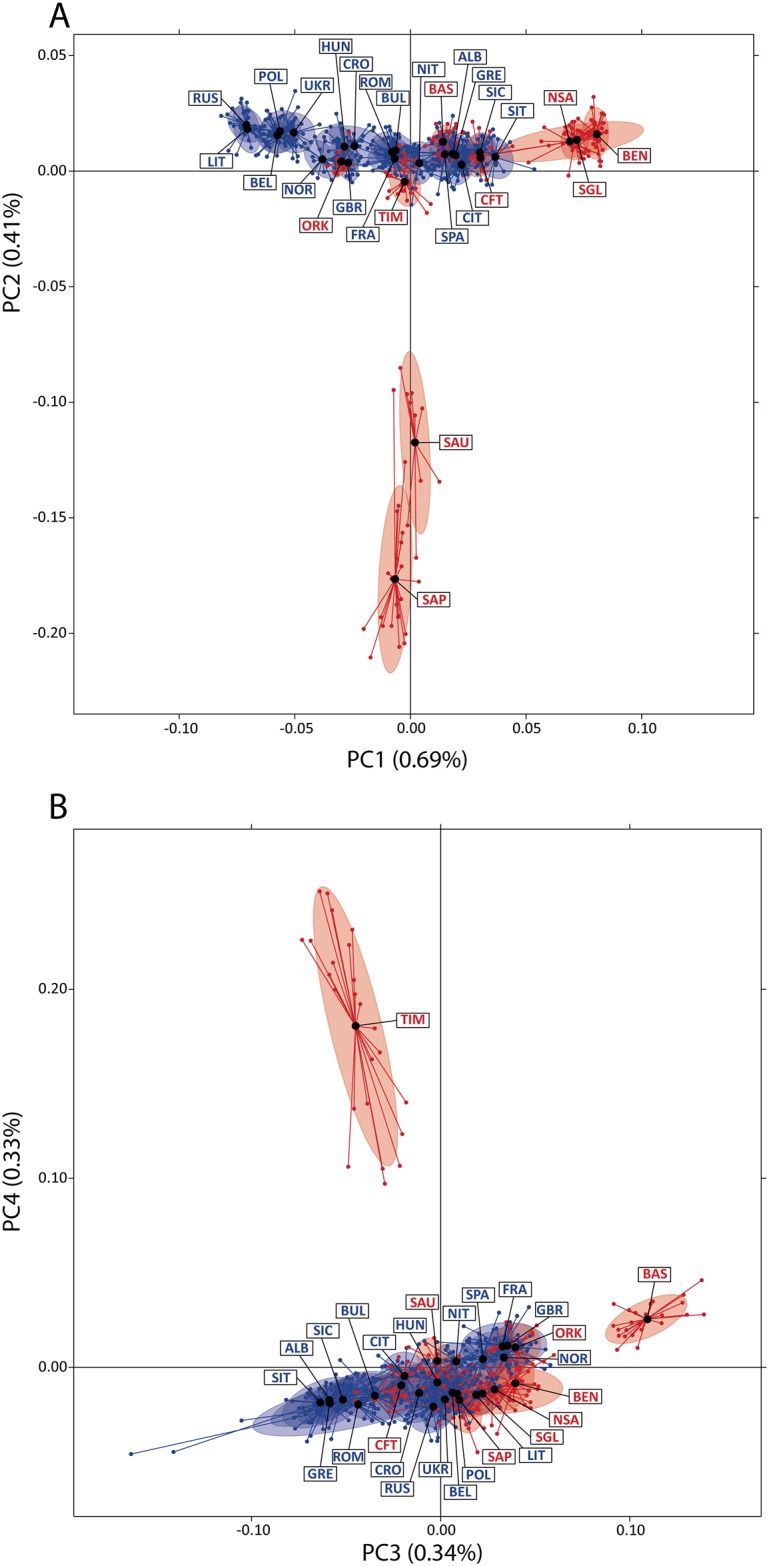
Principal components analysis of the isolated and open populations. (A) Plot of the first and second components and (B) Plot of the third and fourth components. Black dots represent the centroid for each population. Labels as in Table 1.

As a first sight to the results of inter-individual genomic heterogeneity, we plotted a PCA and evaluated the level of scattering within each population (Fig 1). The resulting patterns suggest a non-uniform distribution of inter-individual heterogeneity values. A substantial departure from the common background was observed for seven out of nine isolates: Benetutti, North Sardinia and Sulcis Iglesiente (first component), Sappada and Sauris (second component), Basques (third component) and Timau (fourth component). More importantly for our research question, combining data from the two plots Timau, Sauris and Sappada were the populations showing the highest median distance of individual data from the centroid, followed by Romania and North Sardinia (see S1 Table).

**Fig 2:**
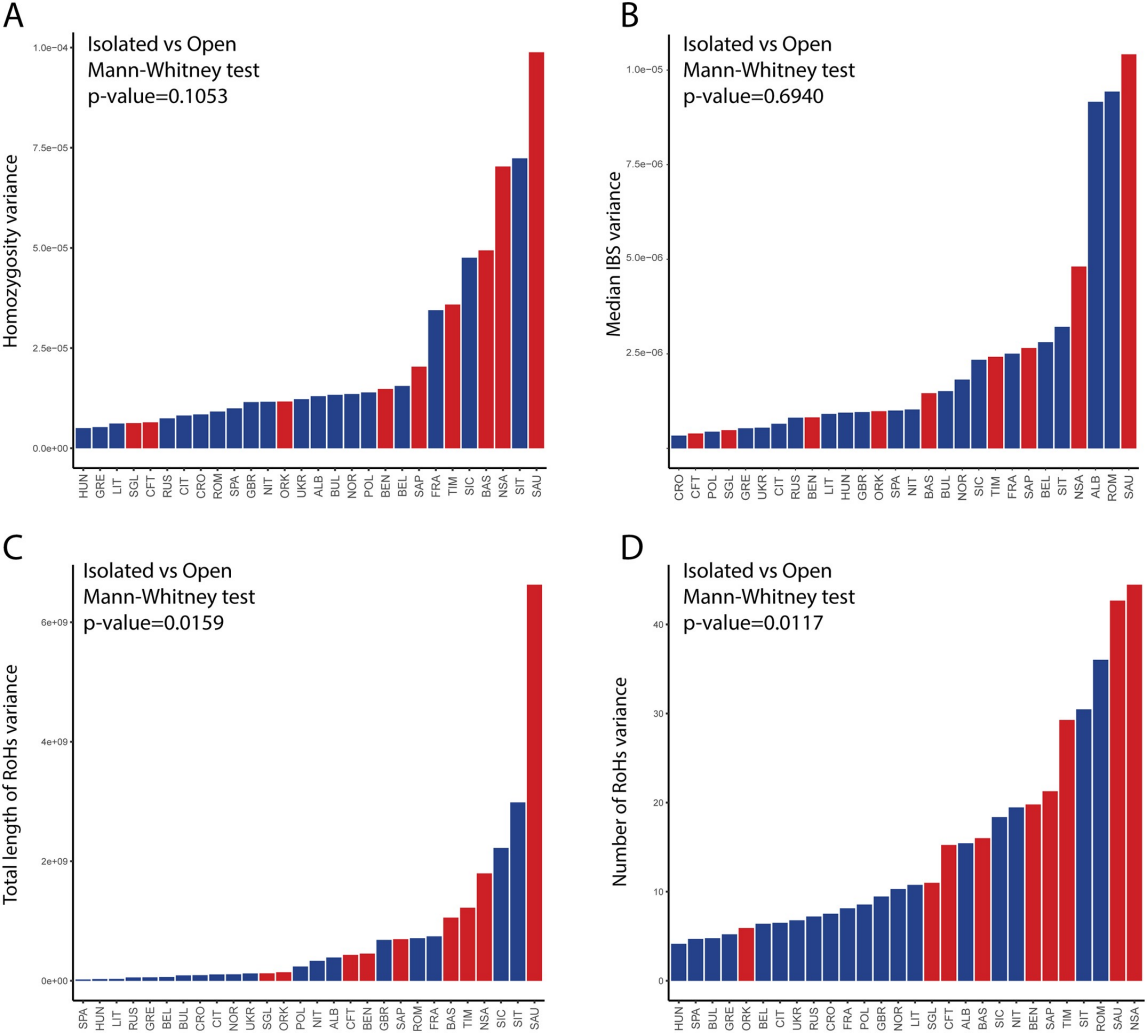
Distribution of inter-individual heterogeneity values across populations and Mann-Whitney U test. Comparison between isolated (red) and open (blue) populations for homozygosity (A), median values of intra-population IBS (B), number of RoHs (C) and total length of RoHs (D).

Thereafter, In order to explore more exhaustively the genomic heterogeneity occurring among individuals within populations, we used four intra-population measures of genomic diversity, based either on single nucleotide (HOM, IBS) or haplotype variation (RoH-KB, RoH-NSEG), for which intra-population variance can be calculated. In contrast with the traditional paradigm of population isolates as genetically uniform entities, taken as a whole isolated populations showed heterogeneity values comparable (HOM, IBS) or higher (RoH-KB and RoH-NSEG; Mann-Whitney test p-value < 0.05) than the open ones (Fig 2). These results were robust to the exclusion of small-sized population sample of Sauris from the dataset (N = 10). Furthermore, given the contribution of Sappada and Timau to the patterns described above, we performed again the comparisons removing also these other two isolated populations. The distribution of values for the open and isolated population groups turned out to be comparable (Mann-Whitney test p-value > 0.05) for all parameters reconfirming that inter-individual comparisons do not support the idea that isolates are structured as genetically uniform entities (S2 Table).

Looking at single populations, the most inbred ones—Sauris, Sappada and Timau—were found to be among the most diverse for all measures along with North Sardinians.

**Fig 3:**
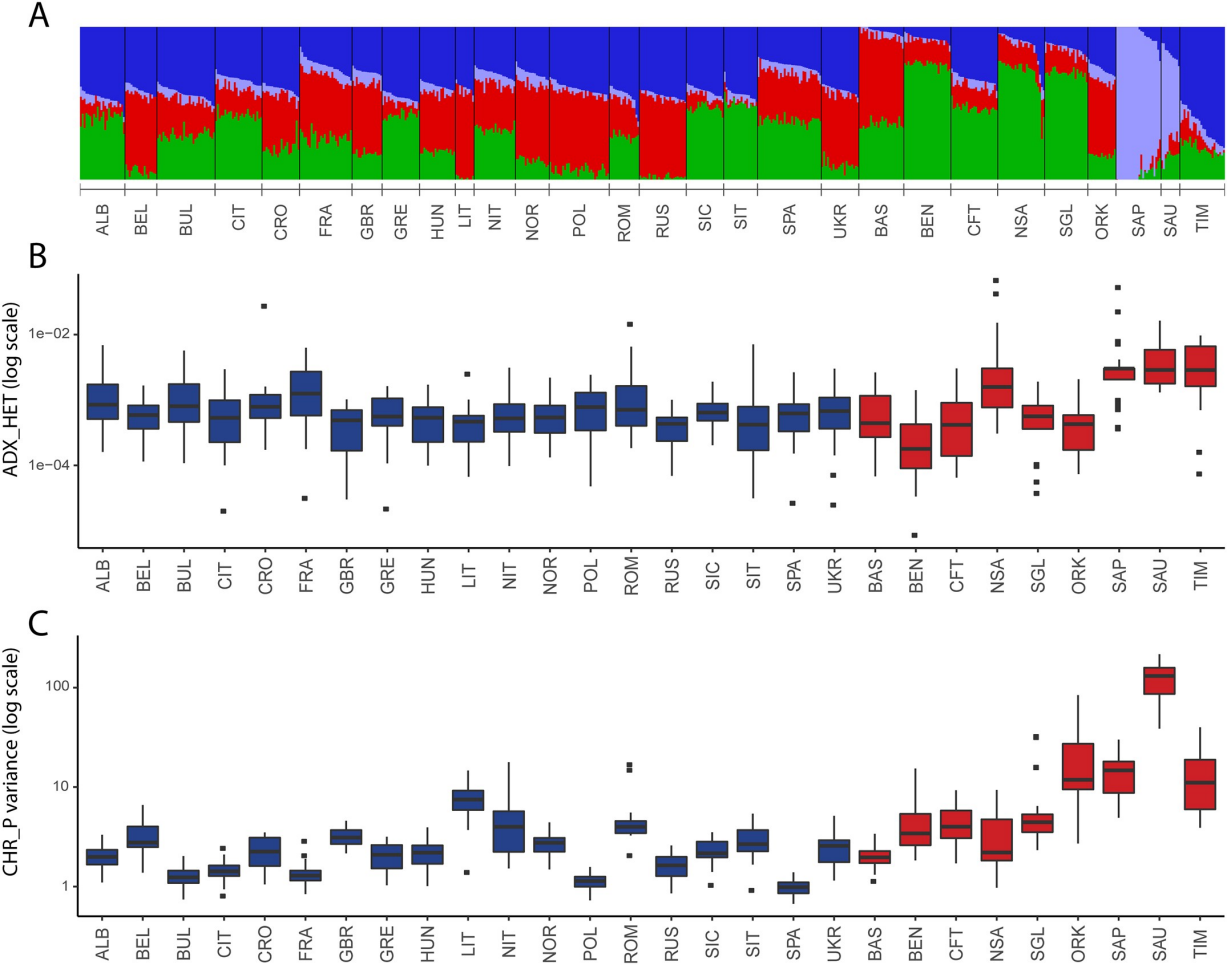
Inter-individual heterogeneity of ancestry components and intra-population haplotype sharing. (A) Maximum likelihood estimates of individual ancestries (K = 4) for the 28 populations under study; (B) intra-population distribution of the admixture heterogeneity measure (y axis log scale); (C) Inter-individual heterogeneities of the total length of chunks among individuals in each population (y axis log scale; see Materials and methods for more detail).

Then, we compared heterogeneity for ancestry proportions (ADX_HET). Also, in this case, isolates, as a whole, were found to be more heterogeneous than open populations (1.38E-03 vs 6.44E-04), but the difference was statistically insignificant (Mann-U-Whitney p-value > 0.05). The greatest values were again obtained in the three population isolates from the eastern Italian Alps, followed by North Sardinians (Fig 3A and 3B), with a noticeable difference: the heterogeneity was more evenly distributed across individuals of the former populations, as indicated by their ratios between average and median values for the best supported K value (K = 4; S1 Fig and S3 Table). Interestingly, we detected a highly prevalent village-specific component in 50% of the genomes from Sappada (12 out of 24, at K = 4) and in 54% of those from Timau (13 out of 24 at K = 5, S2 Fig). The remaining genomes were clearly more heterogeneous, a likely signature of recent admixture.

Finally, we took into account the heterogeneity of the total length of haplotype chunks shared between individuals (CHR_P). The distribution of this parameter reconfirmed the patterns observed for groups (higher values in isolates than open; Mann-Whitney U test based on median variance values, p-value = 0.0029) and single populations (higher values in Sauris, Sappada and Timau). As the only peculiarity, a noticeable signal was provided also from the Orkney islanders (Fig 3C).

**Fig 4:**
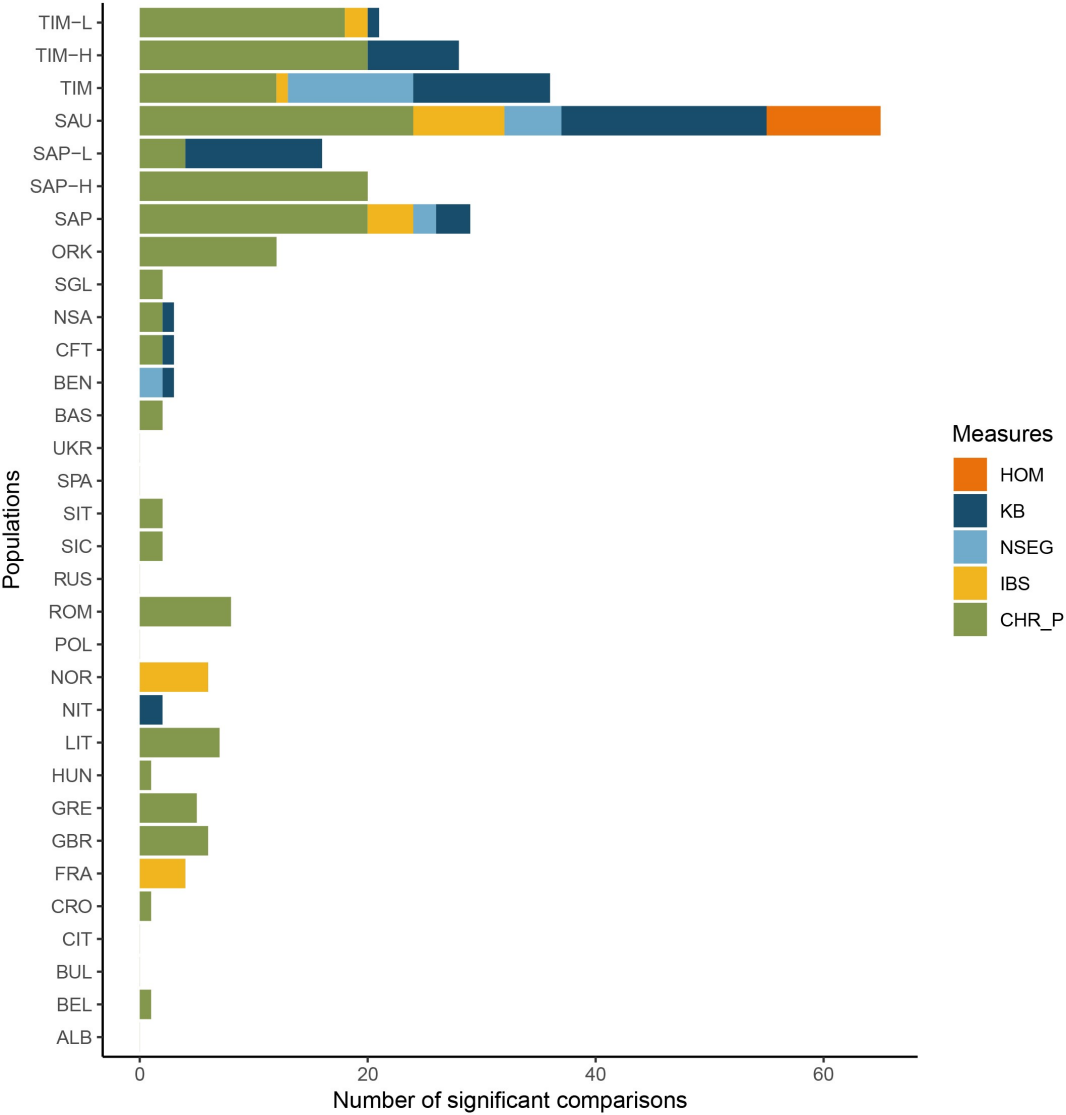
Pairwise comparisons of inter-individual heterogeneity. Number of statistically significant pairwise comparisons with a ratio between standard deviations >1 after Bonferroni correction. For the measures based on pairwise comparisons (IBS and CHR_P), population variance was calculated using the individual median values. Comparisons between Sappada and Timau and their sub-groups (SAP_VSA, SAP_HTA, TIM_VSA and TIM_HTA) were not included.

In order to understand if the results obtained for the three north eastern Italian isolates might be due to introgression of exogenous genetic components, Sappada and Timau samples were splitted into two sub-groups on the basis of ADMIXTURE ancestry proportions (at K = 4 and K = 5 for Sappada and Timau, respectively). In the case of Sauris, sub-groups would had been too small to be separately analyzed. Individuals with a highly prevalent village-specific ancestry (threshold 99%; sub-groups SAP_VSA and TIM_VSA) were taken separate from those with more heterogeneous ancestry, who were termed as SAP_HTA and TIM_HTA. Thereafter, we performed the Levene’s tests for equality of variances between all populations (27 comparisons for all combinations population/measure). Only comparisons with a ratio between standard deviations >1 and significant after Bonferroni correction are shown in Fig 4. The highest number of overall significant comparisons was found for Sauris, which was also the only population with hits in all measures, while the high values of inter-individual heterogeneity for the other north-eastern Italian isolates were not captured by HOM. A relatively high number of significant comparisons still persisted in the HTA groups of both Sappada and Timau, mainly due to KB and CHR_P, respectively. Signatures of inter-individual heterogeneity were recorded also in VSA sub-groups, more evidently in Timau where significant comparisons were observed not only for CHR_P (like in Sappada) but also for KB.

Given the support received by genetic introgression in generating the observed pattern from the analyses described above, we went to infer the time frames of the admixture which likely occurred between SAP_HTA and TIM_HTA sub-groups and geographically-close Italian speaking populations. We preliminarily tested the reliability of our estimates panel using genomic profiles of African-Americans obtained with a much denser SNP set. To this purpose, we retrieved data from the 1000 genomes project phase 3 and used a simple three population model with 30 randomly chosen individuals from the African-American population (ASW) as targets and an equal number of individuals of European (CEU) and African (YRI) origin as sources. Estimates obtained by using our SNP panel and another including 8,142,382 markers (with MAF<0.05) were close each other and consistent with previous results based on molecular data [30]: the admixture event dated at around six generations ago, with an average value across individuals of 6.9+/-3.7 and 6.2+/-2.8 for the high- and low-density SNP sets, respectively (see S4 Table for individual estimates). Then, we applied the same procedure to the admixed sub-groups (SAP_HTA and TIM_HTA) as targets, while the un-admixed ones (SAP_VSA and TIM_VSA) and the northern Italians (NIT) served as sources. The resulting admixture dates were relatively recent, but consistent with the grandfather rule: from 3.8 to 5.5 generations (average = 4.6) in Sappada and from 3.8 to 4.8 in Timau (average = 4.4) (see S5 and S6 Tables for individual results). As a matter of fact, our sample selection criteria proved effective in avoiding sampling of recently admixed individuals, thereby allowing us to draw a picture of the genomic structure preceding the isolation breakdown, an event occurred in the eastern Alps region between the two world wars [30,31].

## Interpretive caveats

The results described above should be interpreted in the light of a number of potential biases and confounding factors. A first issue concerns the adequacy of our SNP panel to represent the genetic variation of the populations under analysis. Although it contains a relatively small number of SNPs compared to those used in other surveys of genomic variation, the GenoChip array should provide an adequate coverage of diversity across European populations due to their large number (92) in the reference panel [19,32]. Furthermore, by implementing the criterion of geographical proximity in our experimental design (see above), we expect to further reduce confounders that could potentially originate from ascertainment bias [33,34].

A second potential issue is related to the likely under-representation of rare alleles in our panel, since they have been shown to retain signals of between and within population differentiation stronger than common alleles [14]. Obviously, increasing the number and type of loci (according to their MAF) and making them proportionate to those occurring in the entire genome (or, better, scanning entire DNAs) leads to more precise evaluations of individual and population genomic structure. However, the estimates of inter-individual genomic heterogeneity in each population should not be significantly influenced by the proportion between common and rare alleles unless it is inconsistently distributed across individuals, as could be the case with stratified populations where sub-populations differ substantially in effective size, gene flow and assortative mating [35].

Third, only one of the parameters used here (intra-population haplotype sharing rate, CHR_P) was estimated using phased data. Despite its relatively low density, the Geno chip has been proved useful to reconstruct informative haplotype chunks after phasing procedure [19]. In order to assess the accuracy of our results, especially for isolated populations, we performed five independent phasing runs and calculated the average individual switch error rate (SER). The average SER was even lower in isolated than open populations (7.51% vs 9.11%), which suggests that the distortion introduced in the estimated length of haplotypes was comparable for the two groups.

Taking into account all these aspects, we believe that our approach is suitable for a preliminary assessment of inter-individual genomic heterogeneity in European populations.

## Discussion

### Inter-individual genomic heterogeneity within European population isolates

Previous GWA studies, which analyzed genetic variation of isolated human populations, focused on measures which summarize single nucleotide and haplotype variation within or among groups (e.g. [11,36,37]). A previous study provided evidence of structure within an isolated population (Cardile, southern Italy [38]), but no comparison with other isolates and open populations was carried out. The possible presence of structure within population isolates is worth exploring in depth since it could be a signature of events of recent admixture and/or subdivision; both could potentially disrupt the homogeneity due to the founder effect and persistence of inbreeding over generations.

To gain new insights into the genomic structure of isolated populations, we decided to focus on the distribution of variance (heterogeneity) of intra-population diversity measures across individuals within populations, rather than relying on their average values. In contrast with their common view as groups of genetically homogeneous individuals, we observed that the inter-individual genomic heterogeneity of isolated populations is at least comparable to that of the open ones. It is worth reminding that applying standard measures of intra-population diversity to our dataset produced the expected pattern, with isolates characterized by higher homozygosity, longer and more numerous ROHs and higher IBS values than open populations, although a clear discontinuity of values between the two groups is not noticeable (see [15]).

Interestingly, three small and highly inbred isolates (Sappada, Sauris and Timau) were characterized by particularly high heterogeneity values, which largely exceeded those calculated in all other populations. Given that there is no evidence to support the presence of sub-groups with distinct matrimonial behaviours for any of them, this finding could hardly be put down to population subdivision. However, the observed patterns could be explained, at least in part, by relatively recent events of genetic introgression, such as those suggested by our admixture dates based on ancestry switches. In fact, after removing the individuals with higher percentages of mixed ancestries from the Sappada and Timau samplings, their number of statistically significant pairwise comparisons for inter-individual heterogeneity diminished substantially (Fig 4). We reason that exogenous components might have survived more easily in the three isolates from northeastern Italy than in other populations for two reasons. Firstly, when most, if not all, matrimonial unions occur within small and highly inbred isolates, as is the case for the three populations cited above, carriers of new genetic components may have a greater chance of contributing to the gene pool. In line with this idea, in our global dataset, a high and significant positive correlation was observed between inbreeding rates (S7 Table) and Admixture inter-individual heterogeneity values (Pearson correlation coefficient: 0.768; p-value<0.001). Secondly, the ratio between sample and census size for Sauris, Sappada and Timau (from 1.8% to 4.8%) is greater than in other isolates (from 1.3% to < 0.1%), which increases the probability of sampling individuals bearing genetic components occurring at low or moderate frequencies.

A retrospective look at previous studies shows that other small-sized European isolates with a very high ratio between sample and census size, namely Clauzetto, Erto, Illeggio, Resia and (another sampling from) Sauris, show a similar pattern to what we observed [36]. A high level of heterogeneity among individuals was in fact evidenced by their ancestry proportions and by the results of different types of principal component analyses (basic, spatial and discriminant). The results obtained were explained by Esko et al. [36] as a signature of population sub-structure. Unfortunately, the data this research work was based on were not released by the authors and, therefore, it was not possible to re-analyze and compare them with our results.

### Implications for association studies

Whatever the cause of this high genomic inter-individual heterogeneity we observed in Sappada, Sauris and Timau, we cannot ignore the question: “what do our results imply for the way in which bio-medical studies are carried out in population isolates?”. Although, the most robust evidence was noticed in some young and small-sized population isolates—which are less used in association studies than the older and larger ones [39]—our results are worthy of attention since they highlight a confounding factor which has not been yet adequately taken into account. In fact, to the best of our knowledge, the effect of increased allelic and haplotypic heterogeneity has been investigated only in relation to the issue of undetected population structure in large scale association studies [40], whereas we argue that it may represent a drawback also for genetic investigations of population isolates.

We suggest that genetic clustering algorithms may be used to test for the presence of individuals with different ancestry proportions within isolated populations, similarly to what has been previously done by Esko et al. [36] (see also [41]). Whenever genomes with substantially more heterogeneous ancestry are detected, it would be worth removing them, re-estimating the parameters of gene-disease association and comparing the new results with those obtained using the whole sample. This could help evaluate whether the genomes with mixed ancestry—in which the reduction of the haplotypic and allelic diversity produced by the effects of the founders and inbreeding should be less detectable—may have acted as confounding factors. For each dataset, different ancestry proportions could be tried as thresholds, and the one able to reduce inter-individual heterogeneity without leading to a significant loss of power should be used.

## Conclusions

In this study we have shed light on the occurrence of relatively high levels of inter-individual heterogeneity in population isolates and proposed a way to monitor their effects on the inferences of association between genes and diseases. This research work challenges the traditional paradigm which considers population isolates as genetically uniform entities, providing further evidence that dichotomizing human populations into open and isolated groups fails to capture the actual relations among their genomic features [15]. We hope that our study can stimulate further investigations based on a wider variety of samples and denser SNP panels or, better, whole genome sequencing, through which a better understanding of the fine-grained genomic structure of human population isolates will finally be reached.

## Supporting information

S1 Fig Cross-validation errors of the ADMIXTURE runs. Average values across the 5 independent replicates at K from 2 to 15.
(PDF)

S2 Fig Maximum likelihood estimates of individual ancestries. Plots from K = 2 to K = 10 for the 28 populations under study.
(PDF)

S1 Table Distances from the centroid. Median and variance distance values were obtained for each population over the PCA plots (first 4 components). Labels as in Table 1.
(XLSX)

S2 Table Mann-Whitney test results. Mann-Whitney U-test p-values comparing variance values between open and all isolated population (first column), excluding Sauris (second column) and excluding all three German-speaking islands (Sauris, Sappada and Timau, third column). Bold numbers indicate significant values (p-value<0.05).
(XLSX)

S3 Table Ratio between mean and median inter-individual heterogeneity. Analysis based on the Admixture components proportion recorded at K = 4.
(XLSX)

S4 Table Date estimates based on ancestry switches inferred with the high- and low-density SNP sets for the 1000 Genomes African Americans. (XLSX)

S5 Table Ancestry proportions, number of ancestry switches and date estimates for the Sappada admixed subgroup. (XLSX)

S6 Table Ancestry proportions, number of ancestry switches and date estimates for the Timau admixed subgroup. (XLSX)

S7 Table Inbreeding coefficient values. Calculated as the proportion of the autosomal genome in runs of homozygosity, excluding the centromeres.
(XLSX)
