# Supplementary figures and images for: Inter-individual genomic heterogeneity within European population isolates
Source: PLoS One. 2019 Oct 9;14(10):e0214564. doi: 10.1371/journal.pone.0214564 (PMC6785074; doi:10.1371/journal.pone.0214564)

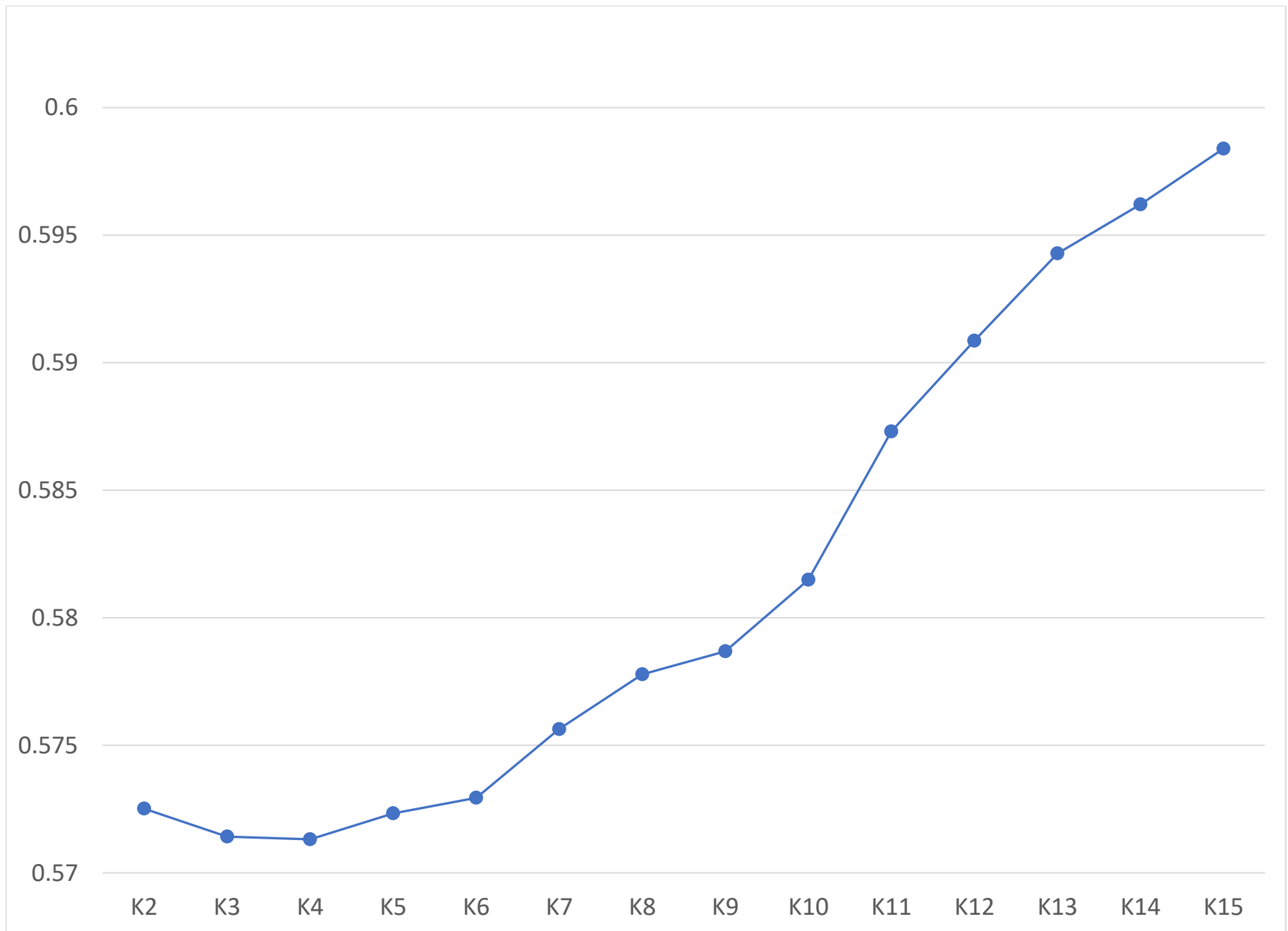

Supplement: S1 Fig — Average values across the 5 independent replicates at K from 2 to 15. (PDF) [file pone.0214564.s001.pdf]

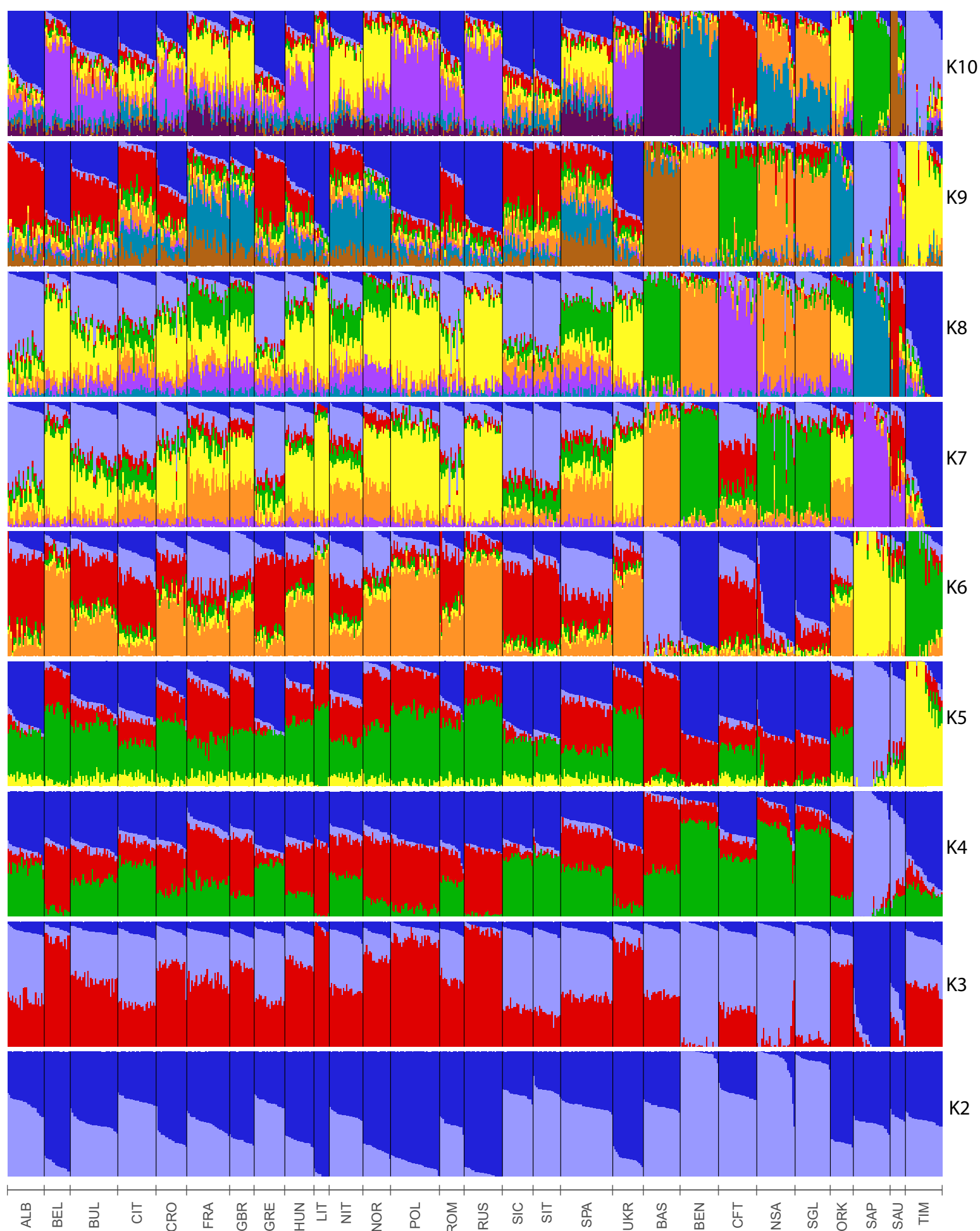

Supplement: S2 Fig — Plots from K = 2 to K = 10 for the 28 populations under study. (PDF) [file pone.0214564.s002.pdf]
